# The Impact of “Death Rattle” on Patients, Informal Caregivers and Healthcare Professionals: A Scoping Review

**DOI:** 10.1177/10499091251382508

**Published:** 2025-09-29

**Authors:** Norah Fagan, Michael Connolly, Nessa Keane, Andrew Davies

**Affiliations:** 1 8870 Academic Department of Palliative Medicine, Our Lady’s Hospice & Care Services, Dublin, Ireland; 2 School of Nursing, Midwifery and Health Systems, 8797 University College Dublin, Dublin, Ireland; 3 Saint Francis Hospice, Dublin, Ireland; 4 Academic Department of Palliative Medicine, Trinity College Dublin, 8797 University College Dublin, Our Lady’s Hospice & Care Services, Dublin, Ireland

**Keywords:** palliative care, terminal care, respiratory sounds, death rattle, psychological distress, scoping review

## Abstract

**Background:**

Audible upper airway secretions (“death rattle”) is a common problem in patients in the last days of life. The objectives of this scoping review was to determine the impact of death rattle on patients, informal caregivers, and healthcare professionals.

**Methods:**

The scoping review used standard methods, and involved searching Medline (Ovid), Embase, CINAHL, PsycInfo, and the Cochrane Library. Studies were included if they reported original data on the impact of death rattle on 1 or more of the specified populations. Hand searching was also undertaken to identify further studies.

**Results:**

Fourteen original studies were identified: 2 studies focused on patients, 7 on informal caregivers, and 5 on healthcare professionals. The impact of death rattle was somewhat variable, although related distress was common amongst informal carers, and also amongst healthcare professionals. The impact of death rattle on patients was less well described, although there was a consensus that death rattle is not distressing to affected patients (but may be distressing to other patients). Various beliefs were associated with distress amongst informal carers, and these may be amenable to educational interventions about the nature of death rattle.

**Conclusion:**

Death rattle is frequently distressing, and strategies need to be developed to mitigate the distress experienced by informal carers, and equally healthcare professionals. Further research is needed to determine the effect on patients experiencing the problem, and also patients witnessing the problem.

## Introduction

Audible upper airway secretions (generally referred to as “death rattle”) are a common problem in patients at the end of life, with a weighted mean prevalence of 35% reported in an earlier review (with a range of 12%–92%).^1^ The term “death rattle” was coined due to its association with imminent mortality,^2^ and whilst many patients develop this problem within the last 24hr of life,^1^ it can occur at earlier timepoints, and can having a somewhat waxing and waning pattern.^3^

The noise (“rattle”) is due to turbulent airflow caused by the upper airway secretions, and is influenced by the amount of secretions, the calibre of the airways, and the rate of breathing.^4^ Bennett suggested 2 distinct subtypes: (a) type 1 – from accumulation of saliva; and (b) type 2 – from accumulation of bronchial secretions.^4^ The pooling of these secretions is worsened by the presence of impaired swallowing, impaired cough reflex, and supine or semi-recumbent positioning.^4^ However, Wildiers et al suggested an alternative classification: (a) “real” death rattle – due to non-expectorated (normal) saliva or bronchial secretions; and (b) “pseudo” death rattle – due to non-expectorated (abnormal) bronchial secretions secondary to aspiration, infection, pulmonary oedema or tumour secretion.^5^

The usual interventions for managing audible secretions involve patient repositioning, oropharyngeal suctioning, and/or the use of anticholinergic medication (eg, glycopyrronium bromide, hyoscine butylbromide).^6^ Another important aspect is informal carer education/reassurance, since death rattle is a frequent cause of distress. Significantly, the relevant Cochrane systematic review concluded that there is little evidence to support any of the aforementioned interventions (non-pharmacological or pharmacological).^7^ Recently, another systematic review re-iterated the relative inefficacy of anticholinergic medication to treat established death rattle (with some new evidence to support prophylactic utilisation).^8^

As discussed, there is general consensus that some informal carers (and others) are distressed by death rattle. The aim (research question) of this scoping review was to determine the exact impact of death rattle on patients, informal carers (family, friends), and healthcare professionals, and so whether alternative strategies need to be developed to manage this common end-of-life phenomenon. An earlier systematic review examined the “impact” of death rattle, but this was undertaken over 10 yr ago, and the review was not focussed on this matter.^1^ A more recent narrative review examined the “experiences” of death rattle, but this involved a limited search of the literature, and the review was again not focussed on this matter.^9^ Hence, there was a need to undertake an up-to-date, comprehensive review of the literature, and it was decided that a scoping review was the optimal format.^10^

## Methods

### Research Question

What is the impact of “death rattle” on patients, informal caregivers and healthcare professionals?

### Design

The methodology employed in this scoping review was based on the framework developed by Arksey and O’Malley,^11^ and subsequent refinements of the original framework.^12,13^ The PRISMA Extension for Scoping Reviews (PRISMA-ScR) was used to guide reporting of the scoping review.^14^

### Search Strategy

Five electronic databases (Medline, CINAHL, Embase, PsycInfo, Cochrane Library) were originally searched in October 2024, and re-searched in February 2025 (to check for any new references). Medline is the National Library of Medicine’s premier database, CINAHL is a database of nursing and allied health literature, Embase is a database of biomedical and pharmacological literature, PsycInfo is a database of behavioural and social sciences literature, and the Cochrane Library is a database of randomised controlled trials and systematic reviews. All databases were searched from inception. The reference lists of all “eligible” full text articles were hand searched for further articles. In addition, relevant chapters in palliative care/medicine textbooks were hand searched for extra articles.

A detailed search strategy was developed for Medline and adapted as needed for the other databases: (Oropharyngeal secretions or airway secretions or bronchial secretions or pulmonary secretions or respiratory secretions or non-expectorated secretions or retained secretions or terminal secretions or respiratory sounds or respiration sounds or noisy respiration or gasping breathing or gurgling breathing or noisy breathing or rattling breathing or terminal breathing or death rattle) and (palliative care or palliat* or hospice care or terminal care or terminal* or end of life care or death or dying).

### Study Eligibility Criteria

Studies were included if they involved adult (>19yr) patients with death rattle, and if the study included information about the related experiences of patients with death rattle, patients without death rattle (ie, witnesses of patients with death rattle), informal carers (ie, family, friends), healthcare professionals, and/or other groups involved in the care of dying patients. Non-English studies were excluded. Studies in non-palliative care settings (eg, intensive care units) were excluded, as were studies focussing on interventions. Case reports, generic review articles, and other records without original information were also excluded.

### Data Management and Synthesis

The EndNote 21™ bibliographic software (Clarivate Analytics LLP, USA) was used to store the retrieved articles, whilst the Covidence systematic review software (Veritas Health Innovation, Australia) was used to screen/manage the retrieved articles. Two reviewers (NF, NK) independently screened the titles and abstracts. A third reviewer (AD) was available to resolve potential conflicts. The same 2 reviewers independently reviewed the full text articles for relevance/inclusion. A third reviewer (MC) was available to resolve potential conflicts. Subsequently, 2 reviewers (NF, AD) independently extracted applicable information from included studies using a review-specific template (ie, study ID, study aim, study population, study methodology, study results). A third reviewer (MC) was again available to resolve potential conflicts. No formal protocol was produced, although the criteria for inclusion/exclusion were explicitly defined within Covidence.

## Results

### Search Results

The search strategy identified 1521 unique references, although most of these were deemed not relevant after assessment (Figure 1). Fourteen primary studies were identified,^3,15–27^ and those with numerical responses are shown in Tables 1-3. Studies were conducted in the United Kingdom (n = 8),^3,20,21,23–27^ in Japan (n = 3),^17,19,22^ in the United States of America (n = 2),^15,16^ and in the Netherlands (n = 1).^18^ Two studies focused on patients,^3,15^ 7 on informal caregivers,^16–22^ and 5 on healthcare professionals.^23–27^

**Figure 1.**
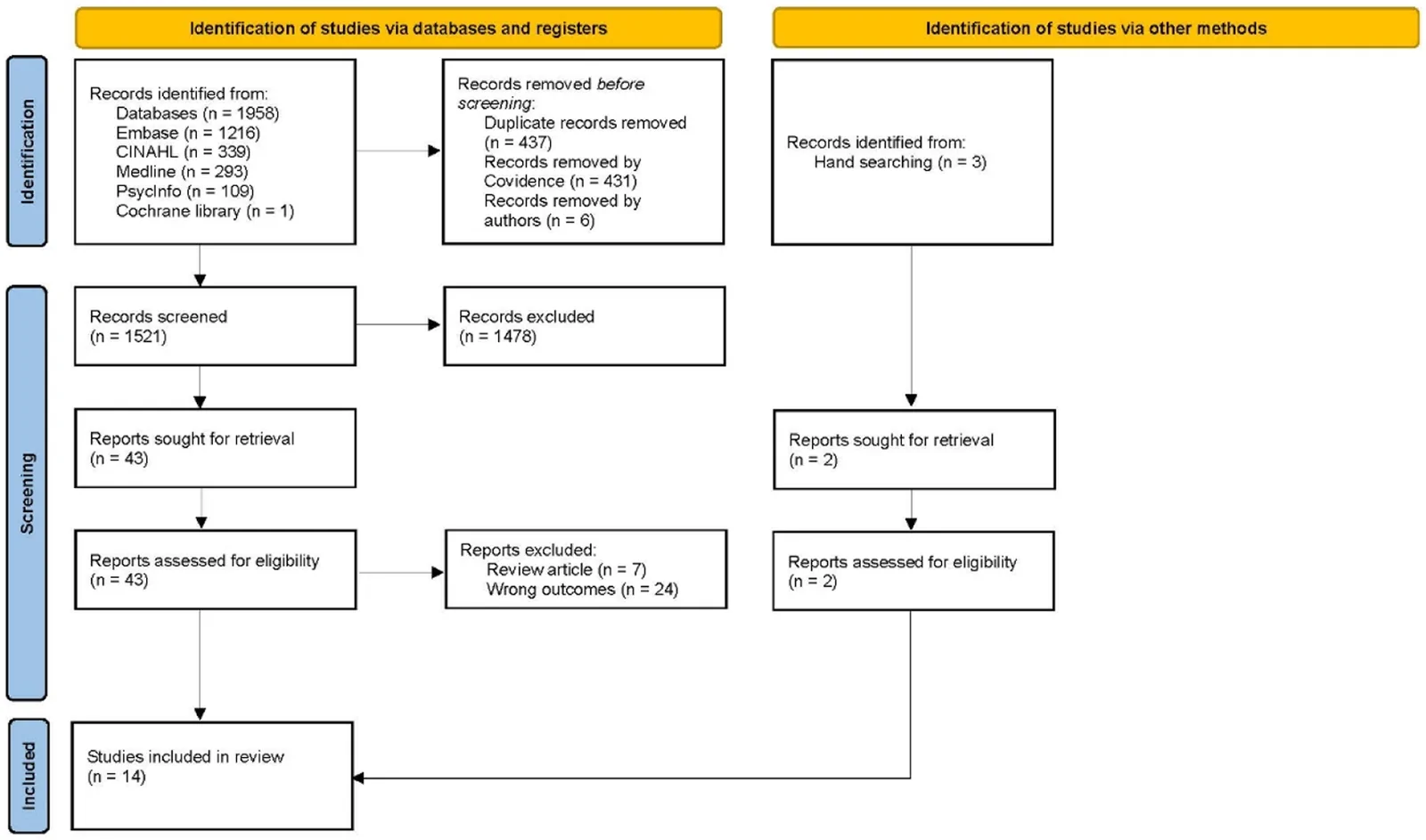
PRISMA Flow Chart

**Table 1.** Patients Experience

Study	Aim	Population	Methodology	Results
Davies et al, 2024^3^ United Kingdom	Secondary analysis of clinical data (including death rattle)	“Cancer patients in the last days of life” (multicentre) n = 200 (182 died during study) Female – 58% Median age – 73yr (range 28-98yr) Cancer diagnosis – 100%	Interventional study (of clinically assisted hydration in last days of life) Assessments (4hrly): Presence of death rattle (yes/no) Modified Richmond Agitation and Sedation Scale/m-RASS)^28^	99/182 (54.5%) developed death rattle Statistically significant association between presence of restlessness/agitation and death rattle in last 48hr of life (*P* < 0.00001)
Campbell et al, 2013^15^ United States America	“To determine if patients with naturally occurring death rattle experience respiratory distress”	“Terminally ill adult patients” (multicentre) n = 71 Female – 42% Mean age – 71yr Cancer diagnosis – 42%	Observation study Assessments (daily): Death Rattle Intensity Scale, Respiratory Distress Observation Scale/RDOS^29^	32 (45%) developed death rattle No statistically significant difference in RDOS scores between patients with and without death rattle

**Table 2.** Informal Caregivers Experience

Study	Aim	Population	Methodology	Results
Oliver et al, 2025^16^ United States America	“To answer…research question…What are the emotions caregivers experience as a result of witnessing the sights and sounds of respiratory changes?”	Bereaved “hospice caregivers” (multicentre) n = 22 Female – 95.5% Mean age – 57.8yr Relationship to patient – spouse 72.5%; child 27.5%	Qualitative study (secondary/sub group analysis)	11 (50%) reported changes in respiratory sounds (not all death rattle) 8 (36%) reported “negative emotional impact” from respiratory changes (not all death rattle)
Shimizu et al, 2014^19^ Japan	“To clarify death rattle-related emotional distress levels among family members”	“Bereaved family carers” (multicentre) n = 390 Female – 46% Mean age – 71yr (+-12yr) Relationship to patient – spouse 45%; child 36%; other 19%	Postal survey (effective response rate 61%) Distress relating to death rattle scale (study specific): 1 = “no distress”; 2 = “a little distress”; 3 = “somewhat distressed”; 4, = “distressed”; 5, = “very distressed”	181 (46%) reported death rattle Distress scale – “no distress” 1.7%; “a little distress” 7.2%; “somewhat distressed” 23%; “distressed” 37%; “very distressed” 29%; missing data 2.2% High distress (“distressed”, “very distressed”) associated with female gender, unawareness that death rattle was a natural phenomenon, fear that patient was drowning, fear that death was imminent, and feeling ”I (carer) was suffocating” [74% carers thought that the patient’s death was imminent; 68% thought that the patient was drowning; 61% worried that the patient might be suffocating; 59% thought that they (carer) was suffocating after hearing the patient’s death rattle for a prolonged period of time; 58% thought that the patient was in pain; 55% thought death rattle was a normal phenomenon; 50% agreed that while they were surprised at first, they became gradually more comfortable’; 13% agreed that they were unable to approach the patient because the rattle frightened them]
Wee et al, 2006a^20^ United Kingdom	“to answer the question: ‘are relatives distressed by hearing the sound of death rattle?’”	“Bereaved relatives” (single centre) n = 27 Female – 78% Relationship to patient – spouse 66.5%; child 15%; other 18.5%	Qualitative study	12 (44.5%) reported experience of death rattle 5/12 (41.5%) expressed negative feelings about death rattle 5/12 (41.5%) expressed neutral or positive feelings about death rattle [Some stated that it was “helpful” in terms of it being a sign of impending death]
Wee et al, 2006b^21^ United Kingdom	“to: confirm or refute our earlier findings that some, but not all, relatives are distressed by hearing the sound of death rattle”	“Bereaved relatives” (single centre) n = 25 Female – 68% Relationship to patient – spouse 60%; child 20%; other 20%	Qualitative study	17 (68%) reported experience of death rattle 10/17 (59%) were “distressed” by death rattle 7/17 (41%) were “not particularly bothered” by death rattle [Some stated that it was “useful” in terms of it being a sign of impending death, and some were more distressed by other aspects of dying] Distress was associated with “distressing connotations”, eg, patient choking, patient drowning
Morita et al, 2004^22^ Japan	“To evaluate the degree to which family members were distressed about…bronchial secretion in the terminal phase”	“Bereaved family members” (multicentre) n = 195 Female – 67% Mean age – 57yr (+-12yr)	Postal survey (effective response rate 65%) Distress relating to death rattle scale (study specific): “not distressed at all”; “not so distressed”; “slightly distressed”; “distressed”; “very distressed”	65 (33%) reported “bronchial secretions” often or very often Distress scale – “not so distressed” 3/65 (4.6%); “slightly distressed” 10/65 (15%); “distressed” 17/65 (26%); “very distressed” 34/65 (52%)

**Table 3.** Healthcare Professionals Experience

Study	Aim	Population	Methodology	Results
Bradley et al, 2010^23^ United Kingdom	“to further our understanding of the factors that influence the decision-making process surrounding death rattle”	“Palliative medicine doctors and clinical nurse specialists” (single centre) n = 15	Qualitative study	6 (40%) reported little or no impact of death rattle on themselves 9 (60%) reported some impact of death rattle on themselves (“ranging from uncomfortable, frustrating or unpleasant to distressing or hard”)
Watts et al, 1997^24^ United Kingdom	“investigate...palliative care nurses’ feelings about the problem...of noisy rattling breathing in dying patients”	“Registered nurses...working in a palliative care unit” (single centre) n = 23	Questionnaire survey (study specific) Assessment: 100 mm visual analogue scales for statements (see results): 0 = “strongly agree”, 100 = “strongly disagree”	“NRB *(noisy rattling breathing)* causes distress to patients” – mean 51.9 mm (+/- 23.4) “Relatives are easily distressed by NRB” – mean 7.8 mm (+/- 6.1) “I have worked with relatives who have been distressed specifically by NRB” – mean 8.8 mm (+/- 5.6) “Death rattle (NRB) in dying patients causes distress to nurses” – mean 28.1 mm (+/- 20.8)

### Patients

The systematic review by Lokker et al concluded the “it is doubtful if patients suffer from this symptom *(death rattle)*”,^1^ although there is little objective evidence to support this assertion. Nevertheless, this is a pervasive opinion within the literature (and within clinical practice).^6,24^ Two studies focussed on the experiences of patients.^3,15^ Campbell et al studied the association with “respiratory distress” (using the Respiratory Distress Observational Scale^29^), and reported that there was no statistically significant difference in respiratory distress compared with patients without death rattle.^15^ However, Davies et al recently reported that in the last 48hr of life, patients with death rattle were more likely to exhibit concurrent restlessness/agitation (based upon the modified Richmond Agitation and Sedation Scale^28^) than patients without death rattle.^3^ In this study, patients were reviewed every 4 hours, and notably only 20.4% reviews that involved death rattle also involved restlessness/agitation.

The literature contains very little evidence about the effect of death rattle on other patients, although the perception is that it is distressing for other patients (and presumably their informal carers).^30^ Indeed, healthcare professionals sometimes prescribe medication to relieve distress in other patients.^23,25^

### Informal Caregivers

Seven studies focused on the experiences of informal caregivers, ie, family members, close friends.^16–22^ The studies included 3 postal surveys of bereaved carers (all from Japan),^17,19,22^ and 4 qualitative interview studies of bereaved carers (two from United Kingdom,^20,21^ one from United States of America,^16^ and one from Netherlands).^18^ Table 2 shows the numerical responses from these studies.

Distress relating to the death rattle was common, but the frequency of distress was variable (41.5%-78%),^20,22^ as was the intensity of the distress.^19,22^ However, some carers were not particularly distressed by death rattle,^20,21^ whilst others developed tolerance/acceptance of the noise over time.^19,21^ Moreover, some carers found comfort in the death rattle as it was a sign of impending death.^20,21^ Of note, some carers reported being more distressed by other symptoms, a decline in cognition, and/or a decline in responsiveness (communication).^18,21^

Of note, Japanese informal carers consistently reported greater distress relating to death rattle than British informal carers.^19–22^ Indeed, many (13%) Japanese informal carers reported that they were “unable to approach the patient because the rattle frightened me”.^19^

Shimizu et al reported that distress amongst Japanese carers was higher in females, in those that did not think that “death rattle was a normal phenomenon” (55% sample), in those that “thought that the patient was drowning” (68% sample), and those that “thought that the patient’s death was imminent” (74% sample).^19^ Similarly, Wee et al reported that British carers that were more distressed if they associated death rattle with “drowning or choking”.^21^ Shimizu et al also reported that distress was higher in those that thought “I *(informal carer)* was suffocating after hearing the patient’s death rattle for a prolonged period of time”.^19^ None of the other studies reported a similar finding. Other factors that have been linked to carer distress are patient distress,^21^ uncertainty relating to patient distress,^18,21^ longer duration of death rattle,^18^ and previous (negative) experience of death rattle.^18^

Health care professionals reported greater distress in the informal carers than in the patients themselves.^24^ Indeed, informal carer distress was often the primary reason for treating death rattle.^23,25–27^

### Healthcare Professionals

Five studies focused on the impact on healthcare professionals,^23–27^ with 1 study including other hospice workers (ie, chaplains, housekeepers, volunteers).^25^ The studies included 1 questionnaire survey (United Kingdom),^24^ and 4 qualitative interview studies of bereaved carers (all from United Kingdom).^23,25–27^ Table 2 shows the numerical responses from these studies.

The impact on healthcare professionals was variable, and ranged from no impact to distressing, but also encompassed helplessness (and frustration) about their ability to manage the problem.^23,27^ Watts et al reported that distress amongst nursing staff was deemed to be more than that in patients, but less than that in informal carers^24^: 100% respondents thought that death rattle was distressing for relatives, 79% that it was distressing for nurses, and 13% that it was distressing for patients.^31^ Indeed, healthcare professionals often prescribe medication to relieve colleague distress/discomfort,^23,25,27^ and to feel that they are doing something (or even to make themselves feel better).^25–27^

## Discussion

This scoping review supports the assertion that death rattle is generally not distressing for the affected patient. However, one study did report an association between death rattle and restlessness/agitation,^3^ although restlessness/agitation was an infrequent occurrence in that cohort of patients. A follow-up study should confirm/refute this association.^32^ Importantly, lack of patient distress is one of the major reasons for contemplating withholding anticholinergic medication.^33^

The literature confirms that death rattle is distressing for many (but not all) informal carers. Certain factors seem to influence distress, and these often relate to a lack of knowledge and/or misunderstanding about the nature of the condition.^19^ Thus, many carers seem unaware that death rattle is an indication that death, that it is a common condition, and that it is not an indication of “choking” or “drowning”, and is generally not distressing for the patient. Surprisingly, given the lack of adequate treatments,^7^ there is a dearth of evidence relating to the use of educational interventions to prevent/relieve informal carer distress. This is an area that requires further investigation.

The literature also confirms that death rattle is distressing for many (but not all) healthcare professionals. In some cases the distress is related to the death rattle itself, but in other cases it is related to helplessness (and frustration) about their ability to manage the problem.^23,27^ It is clear that there is a need for further targeted education for healthcare professionals, and this should include information about the nature of the condition, the options for treatment, and strategies for dealing with distressed informal carers (and colleagues, and other patients). Again, further research is required to identify the optimal methods for providing this education (as there is a current dearth of evidence).

Finally, despite the lack of evidence, healthcare professionals continue to prescribe anticholinergic medication. Many healthcare professionals acknowledge their lack of efficacy, but prescribe them in order to be seen to be “doing something”, as well as to try to limit distress amongst informal carers and other healthcare professionals. Contemporary (evidence-based) guidance on the management of death rattle is required in order to standardise care, and prevent the inappropriate use of medication, and similarly invasive interventions (eg, suctioning).

The strengths of this scoping review are the methods employed, including the broad search of the literature (without time limits). A limitation is that the exclusion of non-English studies, especially as the scoping review identified certain cultural differences relating to the impact of death rattle on informal carers. A further limitation is the paucity of studies specifically assessing distress in patients experiencing death rattle, and equally patients (and their carers) witnessing death rattle.

## Conclusion

Death rattle is a common end-of-life problem, which is associated with distress in informal carers, health care providers, and patients (both those experiencing it, and those witnessing it). Non-pharmacological and pharmacological interventions are relatively ineffective, and so other strategies involving education, as well as emotional support, need to be developed and formally tested.
